# To Readers and Correspondents

**Published:** 1840-12

**Authors:** 


### THE WESTERN JOURNAL.

Vol. II.—No. XII.

LOUISVILLE, DECEMBER 1, 1840.

---

### TO READERS AND CORRESPONDENTS.

This number completes our second volume. The first number of the third, will appear on the first day of January. We thank our friends for their numerous contributions, several of which are now on hand, awaiting an opportunity for publication. As our limits scarcely permit the insertion of all that we receive, we respectfully suggest to those who may favour us with communications to compose them in as condensed a style as possible. Whatever may be our own sins of diffuseness, we ask conciseness from others; beseeching them to follow our precepts rather than our example.

*It must we think be admitted to be possible, indeed, we may affirm, quite easy, with a copious vocabulary of words, and an amplitude and diversity of illustration, ambitiously and profusely poured out, so to obscure a simple or uncomplicated idea, or bury up and deeply inhume, a small fact, that the reader, profoundly and thoroughly mystified and fatigued by his efforts at exhumation and development, shall, at length, endeavour to escape from the circumlocution in which he finds himself involved, by an ornate, laboured and ostentatious style, and in a moment of discouragement or despair, give up, relinquish or renounce the object, to which the annunciation of the*

*author had primitively or originally invited or drawn his attention; and concerning which he might, in the beginning, have felt and cherished a most lively, philosophical, and astute inquisitiveness.* It is, also, possible to exclude from a paper every fact and thought not entitled to admission; to use words of definite meaning, and reduce them to the lowest number compatible with perspicuity; to arrange them with method; to lay down premises clearly and deduce conclusions forcibly; in short, to treat the reader, as a philosopher seeking for knowledge—not as a sciolist satisfied with words. To vary our phraseology, narrative calls for simplicity, and logic for compactness of style—and as every medical paper is made up of one or both these elements, it will, *cæteris paribus*, prove interesting, in proportion to the precision and vigour with which it is written.

